# Behavioral evidence of hunting and foraging techniques by a top predator suggests the importance of scavenging for preadults

**DOI:** 10.1002/ece3.2944

**Published:** 2017-05-01

**Authors:** Antoni Margalida, MªÀngels Colomer, Roberto Sánchez, Francisco Javier Sánchez, Javier Oria, Luis Mariano González

**Affiliations:** ^1^ Department of Animal ScienceFaculty of Life Sciences and EngineeringUniversity of LleidaLleidaSpain; ^2^ Division of Conservation BiologyInstitute of Ecology and EvolutionUniversity of BernBernSwitzerland; ^3^ Department of MathematicsFaculty of Life Sciences and EngineeringUniversity of LleidaLleidaSpain; ^4^ Fundación CDB‐HabitatMadridSpain; ^5^ Boscaje S.L.SegoviaSpain; ^6^ Deputy General Directorate on NatureSpanish Ministry of Agriculture, Food and EnvironmentMadridSpain

**Keywords:** attack techniques, carrion consumption, facultative raptors, habitat quality, kleptoparasitism, Spanish imperial eagle

## Abstract

Scavenging may be a regular feeding behavior for some facultative raptor species occupying low quality habitats and/or with little experience in hunting techniques. However, its importance has been largely underestimated due to methodological limitations in identifying the real proportion in the diet. Here, through direct observations, we assessed the hunting and foraging success of the threatened Spanish imperial eagle *Aquila adalberti* determining the influence of age, sex, breeding status, habitat quality, prey type, and landscape characteristics. From 465 observations, Spanish imperial eagles used hunting in *flight* (42%), *scavenging* (30%), hunting from a *perch* (16%) and *kleptoparasitism* (12%). Our model suggests that *Prey size* and *Prey type* best explain hunting success, followed by *Landscape* and *Sex*. Our findings suggest that Spanish imperial eagles increase hunting success with age, with scavenging and kleptoparasitism regularly used as juveniles. The absence of relationships with any of the variables considered suggests that kleptoparasitism is an opportunistic behavior used sporadically. Scavenging is also independent of habitat quality and landscape characteristics. Accordingly, low prey density is not a driver of carrion use for preadult individuals, suggesting that a lack of hunting ability obliges this age‐class to use this alternative feeding technique regularly. As a result, the threatened Spanish imperial eagle population is also prone to mortality related to the illegal use of poison baits and, potentially, veterinary drugs (i.e., diclofenac).

## 1 Introduction

Carrion consumption is a key ecological process influencing population dynamics, community structure, and ecosystem functioning (DeVault, Rhodes, & Shivik, 2003; Selva & Fortuna, 2007). Carrion resources are ephemeral and extensively used by vertebrates, mainly obligate scavengers (see a review in DeVault et al., 2003, 2016). In terrestrial ecosystems, although obligate scavengers are the main consumers of carrion remains, in the absence of some obligate scavengers such as vultures, a higher use of carrion by facultative scavengers occurs (DeVault et al., 2003; Moreno‐Opo, Trujillano, & Margalida, 2016; Ogada, Torchin, Kinnaird, & Ezenwa, 2012).

For some vertebrate facultative predators, such as raptors, the importance and ecological influence of scavenging has largely been underestimated (DeVault & Rhodes, 2002; DeVault et al., 2003; Sánchez‐Zapata et al., 2010). This is due to the inherent biases of methods used to assess the diet of raptor species, which generally underestimate the presence of carrion and other less conspicuous remains that are difficult to identify (Redpath, Clarke, Madders, & Thirgood, 2001; Margalida, González, et al., 2007; Margalida, Sánchez, González, Oria, & Prada, 2007; Sánchez, Margalida, González, & Oria, 2008). However, several studies have revealed that scavenging is more prevalent than traditional theory implies (Beasley, Olson, & DeVault, 2012; DeVault et al., 2003; Hammerschlag et al., 2016; Wilson & Wolkovich, 2011). The use of camera traps and video‐cameras has provided new and more objective tools to assess the diet of some facultative avian scavengers, demonstrating the importance of carrion in their diet (Moreno‐Opo, Trujillano, Arredondo, González, & Margalida, 2015; Moreno‐Opo et al., 2016; Sánchez‐Zapata et al., 2010) and the important role of facultative scavengers as facilitators of social information transfer leading to nontrophic interactions among species (Kane, Jackson, Ogada, Monadjem, & McNally, 2014; Moleón, Sánchez‐Zapata, Selva, Donázar, & Owen‐Smith, 2014).

According to the Optimal Foraging Theory (MacArthur & Pianka, 1966; Schoener, 1971), predators prefer profitable prey in terms of energy efficiency to maximize fitness, usually net energy intake (Pyke, Pulliam, & Charnov, 1977). However, when the abundance of preferred prey (due to its high profitability) diminishes, the predator can use alternative preys (Steenhof & Kochert, 1988). Under these circumstances, the use of alternative techniques, such as scavenging or kleptoparasitism, can be beneficial to mitigate the absence of prey or low hunting success as a consequence of inexperience (Brockmanhn & Barnard, 1979; Halley & Gjershaug, 1996). Apart from indirect studies on the diet determined through pellet analysis or remains found at nesting sites (Real, 1996; Sánchez et al., 2008), information about scavenging behavior in raptor species other than vulture is scarce and rarely quantified. For example, Sánchez et al. (2008) found that carrion consumption ranged between 2% and 5% according to different diet assessment methods in breeding Spanish imperial eagles *Aquila adalberti*. On the other hand, Sánchez‐Zapata et al. (2010), with the help of automatic cameras, identified golden eagles *Aquila chrysaetos* on 24 (57%) of 42 monitored carcasses, with similar presence throughout the year. This suggests that the role of carrion in the feeding habits of facultative scavenger raptors may be more important than estimated. However, with respect the exposition to pathogens and toxic by‐products of microbial metabolism, obligate scavengers seem have more natural antibodies against botulinum toxin than do facultative scavengers (Blumstein, Rangchi, Briggs, Souza de Andrade, & Natterson‐Horowtiz, 2017; Ohishi, Sakaguchi, Riemann, Behymer, & Hurvell, 1979).

Spanish imperial eagles are distributed throughout the west‐central quadrant of the Iberian Peninsula (González et al., 2008). They are large, highly territorial raptors that feed upon medium‐sized birds and mammals. Most studies about their diet suggest that their main prey is the wild rabbit *Oryctolagus cuniculus* (Ferrer, 1993; González, 1991; Sánchez, Margalida, González, & Oria, 2009; Sánchez et al., 2008). The abundant literature is in contrast with the scarce information available about hunting techniques and feeding behavior (see reviews in Ferrer, 1993; González, 1991; González & Margalida, 2008). In a recent study, which analyzed the size and selection of hunting zones exploited by the species to obtain feeding resources (Fernández, Oria, Sánchez, González, & Margalida, 2009), no additional information was obtained about differences in age classes in hunting techniques or prey selection. Indirectly, it has been documented that the presence of nonadult individuals in breeding pairs implies a lower breeding output (Margalida et al., 2008) and that habitat quality influences breeding success (Margalida, González, et al., 2007; Margalida, Sánchez, et al., 2007; Margalida et al., 2008). Males assume hunting responsibilities and are the main providers of food to the nest, as their smaller body size and other adaptations give them greater agility and maneuverability to improve hunting success (Margalida, Sánchez, et al., 2007). In addition, supplementary feeding for the species (i.e., dead rabbits provided near nesting sites) is a management tool that demonstrates that the species can consume carrion regularly, increasing through this technique breeding success, mainly in low quality territories and pairs including inexperienced (i.e., subadult) individuals (González, Margalida, Oria, & Sánchez, 2006; González, Oria, et al., 2006). Accordingly, scavenging could be a regular feeding behavior for some facultative raptor species occupying low quality habitats and/or when the individuals are inexperienced. Thus, it is necessary to assess the influence of age, habitat quality, and landscape characteristics on the frequency of scavenging in the diet of this species to know their relevance in their trophic ecology.

Given this background, we hypothesized that immature and less experienced individuals (mainly males) can have lower success on hunting techniques and, as a result, use alternative techniques (i.e., kleptoparasitism, scavenging) to optimize foraging success. Here, our goals are to assess the hunting success of Spanish imperial eagles and to determine whether different hunting techniques and foraging success differs according to age, sex, breeding status, habitat quality, prey type, and landscape characteristics. In addition, we estimate the importance of other feeding techniques (kleptoparasitism and scavenging) to obtain food resources, discussing the implications from a behavioral and conservation perspective.

## 2 Methods

### 2.1 Study area

The study was carried out within the framework of the Recovery Plans for this species in the Autonomous Communities of Castilla y León, Castilla‐La Mancha, Madrid and Extremadura. The majority of breeding birds inhabit plains and mountain ranges with patches of Mediterranean forest and “dehesa,” a kind of open forest of *Quercus rotundifolia* and *Q. suber*. Annually, the species' breeding sites were visited at least once a week between the months of January and August (Margalida, González, et al., 2007; Margalida, Sánchez, et al., 2007). Generally the visits started in January, coinciding with the time when the eagles were rebuilding their nests. The monitoring of the territories ended in August, when the chicks left the nest. However, sporadic visits out the breeding period were also carried out coinciding with the monitoring of marked individuals (González, Margalida, et al., 2006; González, Oria, et al., 2006).

### 2.2 General approach

Between January 1990 and December 2013, 465 Spanish imperial eagle hunting attacks (in flight or from a perch), kleptoparasitic actions, or scavenging (feeding behavior) were recorded by direct observation. Observations were made within each area in conditions of good visibility from vantage points. Within study areas, observations were made throughout the entire year. All hunting attacks were recorded during monitoring of nesting territories (*n* = 86) and by observing floater individuals (*n* = 199) in the study area (>2,000 h of fieldwork). All observations were made by the same observer (RS). Observations consisted of making scans noting the predator, the prey, and/or the groups of prey within a radius of 400–500 m around the observer (Altmann, 1974; Kitowski, 2003).

### 2.3 Attack success and foraging success

We recorded behavior as a food‐obtaining attempt (hunting in flight or from perching, kleptoparasitism, or scavenging) when an individual was observed prospecting the territory or perching and performing any of these actions (i.e., hunting techniques in flight or perched, kleptoparasitic interactions or carrion consumption). For each observation, we noted the following information: day, hour, individual, sex, breeding status, habitat quality, habitat characteristics, hunting or feeding technique used, success of the feeding/hunting technique, and prey. We considered a successful attack or kleptoparasitic interaction when the individual captured the prey as a result of the hunting technique used.

A hunting attack was defined as direct rapid flight toward a clearly identifiable prey (Cresswell, 1994), and a capture was an attack that resulted in the raptor catching hold of the prey (Cresswell & Quinn, 2004). Kleptoparasitic actions included interspecific kleptoparasitism (stealing food from other species) and intraspecific kleptoparasitism (stealing food from other Spanish imperial eagles). This feeding technique differs from scavenging because kleptoparasitism involves the opportunistic or habitual stealing of food items from other animals, generally interacting in flight or perching sites, whereas during scavenging, the carrion consumption can be carried out by several individuals that can consume carrion (always in the ground) without interacting among them. For analytical purposes, both were analyzed by pooling as a consequence of limited data. In this way, every hunting attack or kleptoparasitic action was classified as a success or failure. For scavenging behavior, all the observations were considered successful because in all cases the individual accessed the carrion source. All capture attempts with undetermined outcomes were excluded from the analysis (Collopy, 1983).

Overall, 8 of 465 (1.7%) attempts by eagles were of unknown capture success due to distance from the observer and/or local topography. Attacks by individuals whose sex could not be determined were excluded from the analysis (Collopy, 1983).

Eight variables were recorded for each attack (Table 1). The age of the individuals was classified into three categories in agreement with plumages described by González et al. (2008): (1) juveniles from the first and second year, (2) immatures and subadults (“damero,” “damero avanzado,” and imperfect adult) and (3) adults (Table 2). To identify individuals, we took into account the leg‐rings, radio‐tracked individuals, or the characteristics of the plumage (see Bortolotti, González, Margalida, Sánchez, & Oria, 2008; González & Margalida, 2008). In the case of adults, this allowed us to differentiate between breeding and nonbreeding individuals.

**Table 1:** Definition of the variables used in models to analyze attack success and feeding behavior for Spanish Imperial Eagles

SEX: Sex of the individual. Male or female. Nondetermined individuals are not considered
AGE: Age of the individual. Categorical variable with three age‐classes: adults, subadults, and juveniles
ATTACK/FEEDING MODE: Categorical variable with two classes: (1) prospecting flight and (2) from a perch. Kleptoparasitism and scavenging behavior were analyzed separately
PREY SIZE: Prey group size. Discrete quantitative variable (range < 50 gr to >1 kg)
PREY‐TYPE: Corresponds to mammals, reptiles, birds, and other (nonidentified)
BREEDING STATUS: Breeding status of the individual. Breeding vs. nonbreeding
HABITAT QUALITY: Habitat type according to rabbit density. Categorical variable with two classes: high quality vs. low quality
LANDSCAPE: Habitat characteristics according to vegetation structure and humanization. Categorical variable with four types: 1) closed areas (forest formations and shrubland or Mediterranean forest), open areas (without tree or shrub vegetation, and steppe areas, cereal crops, and grasslands), forest‐open *dehesas* (forest areas and areas cleared of vegetation, pastures, and areas of scattered bushes) and humanized areas

**Table 2:** Sexual differences (M: male; F: female) in hunting/feeding techniques used by Spanish imperial eagles according to their age (A: adult; S: subadult; J: juvenile). Percentage appears in brackets

	Flight			Perch			Kleptoparasitism			Scavenging			Total
	A	S	J	A	S	J	A	S	J	A	S	J	
M	37 (52)	68 (61)	8 (16)	16 (22.5)	22 (20)	4 (8)	6 (8.5)	5 (4.5)	11 (22)	12 (17)	16 (14)	28 (55)	233
F	35 (53)	8 (32)	8 (19)	16 (24)	3 (12)	2 (5)	4 (6)	4 (16)	18 (43)	11 (17)	10 (40)	14 (33)	133

The type of attack was defined by the position of the eagle at the beginning of the attack (from a perch or in flight). We categorized two different hunting techniques to obtain food: in flight and from perching. In addition, we separately analyzed the interactions with dead prey. In this case, we analyzed the success of kleptoparasitic actions and the factors (i.e., landscape, habitat quality) affecting scavenging behavior (Table 1).

With respect to the species hunted or fed upon (scavenging or kleptoparasitic actions), prey were grouped into three categories: mammals, birds, and reptiles. These categories were also subdivided according to prey size into six categories including prey of <50, 51–100, 101–300, 301–600, 601–1,000, and >1,000 g.

To determine habitat quality, we used the same methodology as González et al. (2008) considering high quality habitat when more than one rabbit/hectare was found and low quality when the density was below one rabbit/hectare. To categorize the different characteristics of the habitats in which the hunting/feeding took place, we considered the following habitats: closed areas (forest formations and shrubland or Mediterranean forest), open areas (without tree or shrub vegetation, and steppe areas, cereal crops and grasslands), the “dehesas” (forest areas cleared of vegetation, pastures and areas of scattered bushes), and finally humanized areas.

### 2.4 Statistical analyses

Following the methodological approach outlined by Burnham and Anderson (2002), we used the set of variables shown in Table 1 to develop eight a priori hypothesized models to explain attack success. The strategy of selecting a reduced set of candidate models guards against the risk of overfitting and finding spurious relationships (Johnson & Omland, 2004). We used generalized linear models (GLMs) to examine the effects of the selected variables on hunting success. With a GLM, we studied 50 individual kleptoparasitic interactions assessing whether *Sex*,* Age*,* Landscape*,* Habitat,* or *Breeding status* affected the success of kleptoparasitism. Attack success was modeled as a binary variable (1 = success, 0 = failure). We used a logit link function (with binomial error distribution) for attack success models and to assess the effects of sex, age‐class, prey characteristics, breeding status, and habitat features on the attack/feeding mode used as a response variable (see Table 1). The significance of the parameters was studied with the Wald's test.

To determine the model that best explains hunting success, we analyzed the Akaike criteria and the deviance from the model. We followed an information‐theoretic approach in the analyses of our data (Burnham & Anderson, 2002). Model comparisons were based on the bias‐corrected version of Akaike's information criterion (AICc) and were ranked using AICc differences (∆_*i*_) and Akaike weights (*w*_*i*_). We initially tested and compared the base models in Tables 3 and 4. Using the obtained information, we then examined four models (Tables 5 and 6) that included the most important related variables. We did not study quadratic models as we did not have available data. Analyses were performed with the R program (16 R 2.10.1).

**Table 3:** Summary of eight more simple models of success in hunting according to the studied variables. Models are shown, including the size sample (*n*), number of parameters (*K*), AIC correct values, Δ*i* = AIC*c* –AIC_c_ *min* values and Akaike weights (*wi)*, based on AIC*c*. Models are ordered in terms of Δ*i* for AIC*c*

Model	*n*	*K*	AICc	Δ*i*	*W*_*i*_
$\theta_{Sex}$	228	3	313.81	0.00	1.00
$\theta_{Prey\;size}$	264	9	347.86	34.05	0.00
$\theta_{Prey\;type}$	268	4	352.61	38.80	0.00
$\theta_{Age}$	268	4	365.73	51.92	0.00
$\theta_{Landscape}$	268	7	369.27	55.46	0.00
$\theta_{Habitat\;quality}$	267	3	371.46	57.65	0.00
$\theta_{Breeding\;status}$	267	4	372.13	58.33	0.00
$\theta_{Technique}$	268	3	373.42	59.61	0.00

**Table 4:** Summary of eight more simple models of success in hunting techniques according to the studied variables. Models are shown, degrees of freedom (*df*), Deviance and significance level model (*p*‐value)

Model	*df*	Deviance	*p*‐value
*Sex*	1	5.75	.017
*Prey size*	7	30.67	<.0001
*Prey type*	2	15.36	<.0001
*Age*	2	3.40	.183
*Landscape*	2	9.80	.007
*Habitat quality*	1	0.43	.512
*Breeding status*	1	0.04	.832
*Technique*	5	12.54	.028

**Table 5:** Summary of the four models obtained by combining the most significant variables. Models are shown, including the sample size (*n*), number of parameters (*K*), AIC correct values, Δ*i* = AICc−AIC_c_min*,* values, and Akaike weights (*wi)*, based on AIC*c*. Models are ordered in terms of Δ*i* for AIC*c*

Model	*n*	*K*	AICc	Δ*i*	*W*_*i*_
$\theta_{sex+Prey\;size+Prey\;type+Landscape}$	224	13	274.23	0.00	0.55
$\theta_{Prey\;size+Prey\;type+Landscape}$	224	12	274.74	0.51	0.42
$\theta_{Prey\;type+Prey\;size}$	224	9	280.18	5.95	0.03
$\theta_{Prey\;size+Landscape}$	224	10	288.94	14.71	0.00

**Table 6:** Summary of the four models obtained by combining the most significant variables. Models are shown, degrees of freedom (*df*), deviance, and significance level model (*p*‐value)

Model	*df*	Deviance	*p*
$\theta_{sex+Prey\;Size+Prey\;type+Landscape}$			
Prey type	2	14.339	.0008
Sex	1	7.2637	.0070
Prey size	5	26.9652	<.0001
Habitat	3	12.5862	.0056
$\theta_{Prey\;size+Prey\;type+Landscape}$			
Prey type	2	14.339	.0008
Prey size	5	31.976	<.0001
Habitat	3	12.082	.007
$\theta_{Prey\;type+Prey\;size}$			
Prey type	2	14.339	.0008
Prey size	5	31.976	<.0001
$\theta_{Prey\;size+Habitat\;quality}$			
Prey size	5	31.4722	<.0001
Habitat	3	8.2713	.041

## 3 Results

Spanish imperial eagles obtained food through: hunting in *flight* in 194 (41.7%) of the cases, hunting from a *perch* in 73 (15.7%), *kleptoparasiting* in 57 (12.3%), and *scavenging* in 141 occasions (30.3%).

### 3.1 Influence of age‐class on feeding behavior and hunting techniques

We found significant differences among age‐classes on the feeding techniques used (χ^2^ = 110.05, *df* = 6, *p* < .0001). Juveniles used *scavenging* (61%) more frequently than adults (16.3%) and subadults (22.7%). In addition, juveniles used *kleptoparasitism* (61.4%) more than adults (19.3%) and subadults (19.3%) (Table 2). On the contrary, the technique more frequently used by adults was hunting in *flight* (52.6%) followed by hunting from a *perch* (23.4%) and s*cavenging* (16.8%). In the case of subadults, hunting in *flight* was also the most used technique (56.7%) followed by *scavenging* (19.4%) and hunting from a *perch* (17.2%).

### 3.2 Sexual differences in feeding behavior and hunting techniques

We identified the sex in 366 individuals. No intersexual differences were found in the techniques used by adults (χ^2^ = 0.32, *df* = 3, *p* = .96) and juveniles (χ^2^ = 6.21, *df* = 3, *p* = .10, Table 2) but we did find differences in subadults (χ^2^ = 14.87, *df* = 3, *p* = .002). Subadult females used *scavenging* more frequently (40% vs. 14.4%), whereas subadult males used hunting in *flight* more frequently (61.3% vs. 32%).

### 3.3 Modelling success in hunting in flight vs. from a perch

Of the eight models examined (Tables 3 and 4), according to the Akaike criteria, the best factor included the variable $\theta_{Sex}$, which indicates that attack success is mainly influenced by the *Sex* of the individual, with females being more successful than males (females: 58.9%; males: 41.06%). Taking into account the deviance of the models, *Prey size* and *Prey type*, together with *Landscape*, explain an important variability in hunting success. Table 4 shows how, among the eight variables considered, only three do not show significant results to explain their influence on hunting success (*Age*,* Habitat,* and *Breeding status*). In contrast, *Sex*,* Prey size*,* Prey type*,* Landscape,* and *Technique* showed significant results.

Based on the Akaike values and the deviance, we studied four new models (Tables 5 and 6). *Prey size* and *Prey type* are the variables that best explain hunting success, followed by *Landscape* and *Sex*, which only explained 7% of the variability. Using the parsimonious principle, we can accept that the model takes into account prey characteristics (*Prey size* and *Prey type*) and *Landscape*. In this sense, the most success occurs with reptiles (100% success) and prey of <50 gr (89% success). With respect to landscape characteristics, hunting success in closed areas was 35%, but increased until 50% in the remaining areas (open areas, dehesas).

### 3.4 Modelling kleptoparasitism interactions

Of the 57 individual kleptoparasitic interactions observed, 82.45% were successful. *Sex*,* Age*,* Landscape*,* Habitat,* or *Breeding status* affected the success of kleptoparasitic actions, but none of the variables were significant (Table 7).

**Table 7:** Summary of the five models obtained by combining the most significant variables of success in kleptoparasitic interactions according to the studied variables. Degrees of freedom (*df*), deviance (Dev), and significance level model (*p*‐value)

	*df*	Dev. res.	*df* res.	Dev	χ^2^
Null			41	43.65	
*Sex*	1	0.05	40	43.60	0.83
*Age*	2	0.23	38	43.37	0.89
*Landscape*	5	2.86	33	40.51	0.72
*Habitat quality*	1	0.03	32	40.48	0.88
*Breeding status*	1	0.02	31	40.461	0.89

## 4 Discussion

Spanish imperial eagles, as in other raptor species including the genus *Aquila*, use different attack techniques, with hunting from the air and from perching sites being the most common techniques (see Cresswell & Quinn, 2010; Martínez et al., 2014; Quinn & Cresswell, 2004). However, our findings also show that kleptoparasitism and scavenging are a regular feeding behavior, mainly in juvenile age‐classes. Kleptoparasitism involves the opportunistic or habitual stealing of food items from other animals (Brockmanhn & Barnard, 1979). This results in fitness benefits for the kleptoparasite by saving time and effort (Brockmanhn & Barnard, 1979; Cresswell, 2004; Shealer, Spendelow, Hatfield, & Nisbet, 2005). According to our results, Spanish imperial eagles increase their hunting success with age, with scavenging and kleptoparasitic actions being important alternatives techniques to obtain food during the juvenile stage (first and second year). The inexperience of juvenile individuals obliges them to adopt these alternatives to obtain prey and save time and effort (Shealer et al., 2005). However, some individuals seem to use scavenging throughout their life regardless of habitat quality, suggesting that individual personality also could influence this behavior (authors unpubl. data). These individuals can learn to handle these less valuable prey as alternative prey (Hughes, 1979), balancing the biomass provided by other prey, as well the time and energy invested to obtain them with hunting techniques.

Several studies have shown that hunting success is higher in adults than in preadult individuals (Toland, 1986; Redpath, Amar, Madders, Leckie, & Thirgood, 2002; Kitowski, 2003). In the case of Spanish imperial eagles, until individuals achieve enough experience, preadults use kleptoparasitism and scavenging to obtain food resources. The fact that none of the variables adequately explained the success of kleptoparasitic interactions suggests that this is an opportunistic behavior that individuals use sporadically. With respect to the use of scavenging, the important frequency of carrion consumption is unexpected for a species with a diet mainly based on live prey (González, 1991; Sánchez et al., 2008), although methods to assess the diet could also influence biases in their determination. According to our results, the absence of a relationship to landscape variables, such as habitat quality, suggests that scavenging behavior is independent of the abundance of live prey. In this sense, it should be noted that the estimates of habitat quality (i.e., density of rabbits) may not be completely reliable due to methodological limitations (Fernández de Simón et al., 2011). Moreover, prey abundance does not imply prey availability (Ontiveros, Pleguezuelos, & Caro, 2005) nor is the proportion of prey consumed selected in proportion to its occurrence (Margalida, Bertran, & Heredia, 2009). On the other hand, on several mammalian carnivores have been suggested the switch between hunting and scavenging based on seasonality in carrion supply and prey vulnerability to predation (Pereira, Owen‐Smith, & Moleón, 2014). According to our models, low prey density is not a driver of carrion use for preadult individuals, suggesting that poor hunting abilities obliges this age‐class (mainly juveniles) to use this alternative feeding behavior regularly. Although to our knowledge this is the first quantification of the importance of scavenging behavior in a larger eagle through direct observations, a similar result was indirectly observed in golden eagles in a study with cameras and carrion provided experimentally. Golden eagles appeared at 54% of the carrions observed (Sánchez‐Zapata et al., 2010). In addition, although Spanish imperial eagle is generally considered to be specialized in the capture of rabbits (Ferrer & Negro, 2004), recent studies detected an important variability in the diet spectrum found between regions (Sánchez et al., 2009). This suggests that when the main prey (i.e., wild rabbit) is scarce, the Spanish imperial eagle's diet is based on alternative prey, such as pigeons or carrion. These dietary shifts were probably accentuated after the rabbit crash between 1983 and 1985 as a consequence of the viral hemorrhagic disease (RHD), partially modifying the Spanish imperial eagle's dietary habits (Sánchez et al., 2009).

According to our model, prey characteristics and, to a lesser degree, landscape, determine the hunting success of Spanish imperial eagles. This agrees with several studies that have shown that the hunting strategies, prey type, and prey vulnerability may also be factors determining hunting success (Cresswell & Quinn, 2004; Kenward, 2006; Cresswell, Lind, & Quinn, 2010). Our models show that sex is not a relative good predictor (7%) of hunting success in Spanish imperial eagles, which is similar to the results obtained in other studies with golden eagles (Collopy, 1983), African peregrines *Falco peregrinus minor* (Jenkins, 2000) and Bonelli's eagles *Aquila fasciata* (Martínez et al., 2014). However, habitat quality and prey type and size are important predictors. Thus, the openness of the habitat is a determining factor explaining hunting success for terrestrial prey, with the maintenance of open landscapes being an important management action to improve hunting opportunities (Martínez et al., 2014; Ontiveros et al., 2005).

This approach shows the importance of direct observations to increase our knowledge of individual behavioral traits that can contribute to improve management and conservation actions. For example, with respect to the important role of scavenging on preadult individuals, this finding suggests that Spanish imperial eagles are also prone to mortality related to the illegal use of poison baits, as occurs with other vulture species (Hernández & Margalida, 2008, 2009; Margalida et al., 2014; Mateo‐Tomás, Olea, Sánchez‐Barbudo, & Mateo, 2012). In fact, between 1990 and 2010, a total of 117 Spanish imperial eagles were poisoned (Margalida, 2012), suggesting that this is a key factor for Spanish imperial eagle conservation (González, Margalida, Mañosa, Sánchez, & Oria, 2007). A more recent threat related to scavenging behavior is the ingestion of livestock carrion treated with diclofenac, a nonsteroidal anti‐inflammatory drug (NSAID), which was the main cause of vulture population declines in Asia (Green et al., 2004; Oaks et al., 2004) and was authorized in Spain as of 2013 (Green, Donázar, Sánchez‐Zapata, & Margalida, 2016; Margalida et al., 2014). The potential threat to facultative raptors was evidenced by the mortality of Steppe eagles *Aquila nipalensis* (Sharma et al., 2014) suggesting the possibility that diclofenac is toxic to other accipitrid raptors, such as the threatened Spanish imperial eagle.

## Conflict of Interests

None declared.
